# Alien eating alien - rapid spread of *Aceria fraxiniflora*, a non-native gall mite of the invasive green ash (*Fraxinus pennsylvanica*) in Central-Eastern Europe

**DOI:** 10.1007/s10493-023-00849-5

**Published:** 2023-10-11

**Authors:** Márton Korda, Géza Ripka, Karel Hradil, Milka Glavendekic, Dinka Matosevic, Boris Hrasovec, Márton Paulin, Anikó Hirka, György Csóka

**Affiliations:** 1 Faculty of Forestry, Institute of Environment and Nature Protection, University of Sopron, Sopron, 9400 Hungary; 2 Directorate of Plant Protection and Oenology, National Food Chain Safety Office, Budapest, 1118 Hungary; 3 Central Institute for Supervising and Testing in Agriculture, Jicin, 506 01 Czech Republic; 4 Faculty of Forestry, University of Belgrade, Belgrade, 11030 Serbia; 5 Department for forest protection and game management, Croatian Forest Research Institute, Jastrebarsko, 10450 Croatia; 6 Faculty of Forestry and Wood Technology, Institute of Forest Protection and Wildlife Management, University of Zagreb, Zagreb, 10000 Croatia; 7 Forest Research Institute, Department of Forest Protection, University of Sopron, Mátrafüred, 3232 Hungary

**Keywords:** Eriophyid mite, Green ash, Invasive alien species, Passive spread, Potential biocontrol

## Abstract

The North American gall mite *Aceria fraxiniflora* was first recorded in Europe in southeast Hungary in 2017. Since then, it has shown a remarkably rapid spread on its host, the also North American green ash (*Fraxinus pennsylvanica*). By the beginning of 2023 it has been recorded in eight Central-Eastern European countries. In 2022 it was recorded on the other North American ash (*Fraxinus Americana*) in Zagreb (Croatia) and in Szarvas Arboretum (SE Hungary). Possible reasons and outcomes of this spread are discussed.

## Introduction

**Fig. 1:**
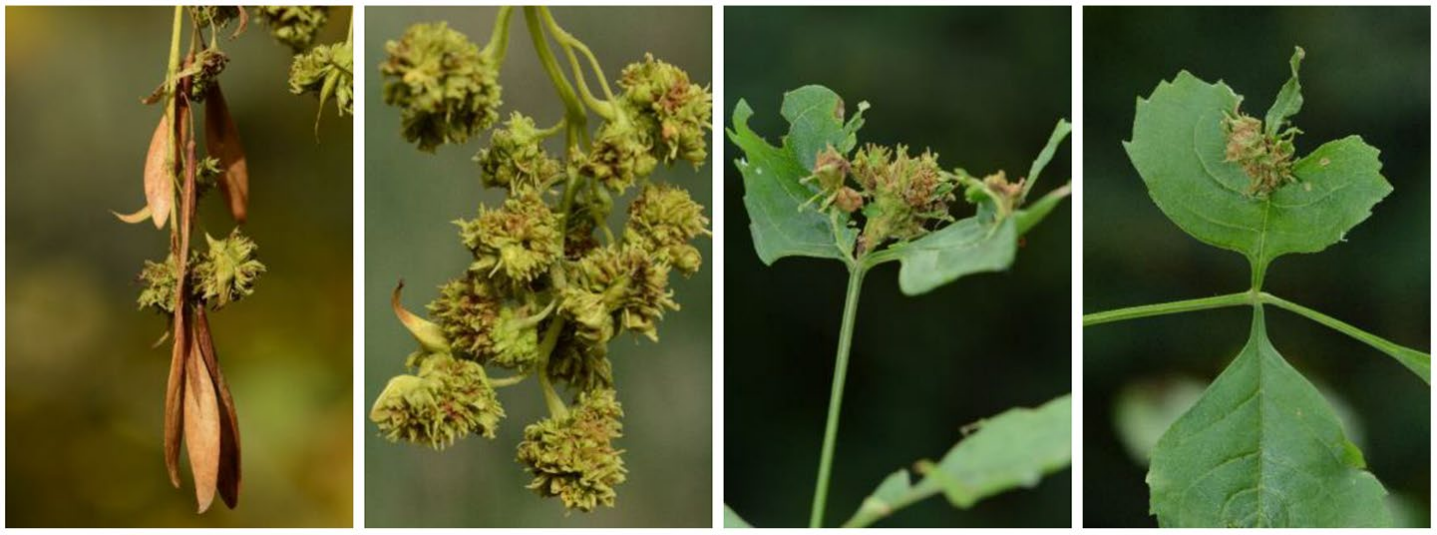
Galls of the non-native *Aceria fraxiniflora* on fruits (left) and leaves (right) of the also non-native green ash (near Gyomaendrőd, SE Hungary) (Photos: Márton Korda)

Eriophyoid mites (Acari: Eriophyoidea) are among the smallest herbivorous arthropods. Approximately 1,200 species have been recorded from Europe (Dr Enrico de Lillo, pers. comm.). They all feed on the saps of the above ground parts of a wide variety of host plants. The largest genus, *Aceria*, includes more than 1,000 species worldwide (Elhalawany et al. 2018). In the fauna of Europe 346 *Aceria* species are known, 145 of which develop on woody plants (Amrine & de Lillo unpubl. database). On the European ashes (*Fraxinus* spp.) six native eriophyoid species have been recorded. The most common species of these six is *Aceria fraxinivora* (Nalepa) causing cauliflower-like galls on flowers and fruits, and the leaf-rolling *Aculus fraxini* (Nalepa). A gall mite of North American origin, *Aceria fraxiniflora* (Felt), new to the European fauna, was recorded in Hungary in 2017 (Korda et al. 2019). On top of causing galls on flowers and fruits it induces tissue proliferation on shoots, leaves and leaf stalks as well (Fig. 1).

Its host, the also North American green ash (*Fraxinus pennsylvanica* Marshall) was introduced most likely to the UK in the second half of the 18th century (Rehder 1927; Scheller 1977), so it has been present in Europe for more than 2.5 centuries. First it was planted in parks, arboreta and botanical gardens, but from the second half of the 19th century foresters showed an increasing interest, and therefore planting experiments were initiated particularly in Germany and Hungary (Fekete 1877; Marosi 1884; Schmiedel 2011). At the turn of the 19th and 20th centuries it was widely planted Europe-wide, mainly in floodplains (Schmiedel 2011; Drescher and Prots 2016; Korda 2018). Its ability for spontaneous mass regeneration and effective spread became conspicuous in the 1920s (Korda 2018). Now it is widespread in almost all of Europe and causes significant problems both from the forestry and natural conservation point of view (Scheller 1977; Schmiedel 2011; Prots et al. 2011, Pysek et al. 2012, Drescher and Prots 2016, Kézdy et al. 2017; Korda 2018, GBIF 2022), particularly on floodplain meadows, in groves and swampy areas (Török et al. 2003; Csiszár and Bartha 2008; Schmiedel 2011; Khapugin 2019). Any chemical control is excluded in protected areas particularly in floodplains. Only mechanical control may be used widely (removing the seed-producing trees and seedlings, cutting the sprouts). However this kind of control is expensive, and time and labour demanding (Csiszár and Korda 2017).

Hardly any herbivorous arthropods are known to feed on green ash, i.e., two scale insects (Hemiptera: Coccoidea), *Parthenolecanium corni* (Bouché) and *Pseudaulacaspis pentagona* (Targioni-Tozzetti), on its shoots and twigs (Ripka et al. 1996). Both are widely polyphagous species. They may be found commonly and are often abundant on deciduous, ornamental, forest and fruit trees and shrubs belonging to many families (Kozár 1998). Also worth mentioning is an eriophyoid mite, *Aculus epiphyllus* (Nalepa), living near the veins on the underside of the leaves (Ripka et al. 2020). A North American aphid, *Prociphilus fraxinifolii* (Riley) (Hemiptera: Aphididae), forming ‘leaf-nest’ pseudogalls, was first found in Hungary in 2003 (Remaudière and Ripka 2003; Ripka 2005). None of these species has shown any significant impact on green ash’s fitness or its fecundity.

The aim of this study is to provide an updated distribution map of *Aceria fraxiniflora* and initiate international interest concerning the impact – as a potential biocontrol agent – on its invasive host-plant, *F. pennsylvanica*.

## Methods

We looked for and identified the mites based on the characteristic galls they cause on the green ash. The determination was based on the description (Korda et al. 2019). Data were collected throughout the year, as the gall often remains (at least partially) for the winter, and can be easily and clearly identified. The identification of ash individuals was based on Fitschen (2002).

Distribution data were collected by the authors in city alleys, roadside trees and green ash stands in eight Central Eastern European countries. After detection of galls, fresh galls were taken to the laboratory to identify the causing organism under the microscope. The mites are morphologically one species and identical to the species described in Hungary. Additional records were provided by other persons – see the acknowledgements. The coordinates of the collection sites were recorded and used to create a map edited by QGIS v.3.16.11.

## Results

**Fig. 2:**
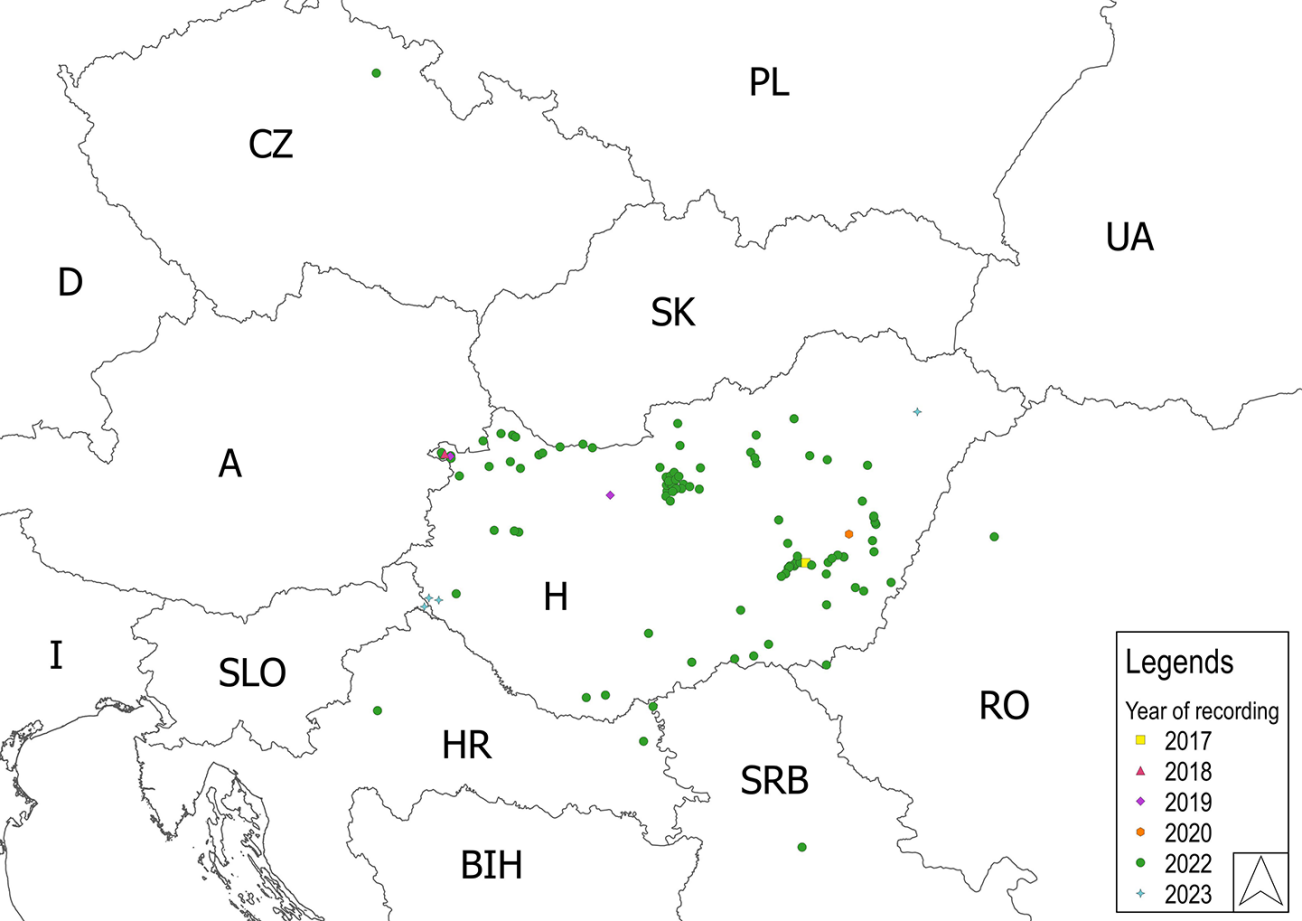
Presently known distribution of *Aceria fraxiniflora* in Central Eastern Europe. Abbreviations: A: Austria, BIH: Bosnia and Herzegowina, CZ: Czech Republic, D: Germany, HR: Croatia, H: Hungary, I: Italy, PL: Poland, RO: Romania, SRB: Serbia, SLO: Slovenia, SK: Slovakia, UA: Ukraine

Positive distribution records have been collected in eight Central Eastern European countries (Table 1; Fig. 2). All records in Table 1 refer to *F. pennsylvanica* as host. More details concerning the Hungarian distribution can be found in Korda et al. (2022). In 2022 *Aceria fraxiniflora* was recorded on white ash (*Fraxinus americana* L.) in Zagreb (Croatia; 45°47.34’N, 15°59.69’E) and in the Szarvas Arborétum (SE Hungary; 46°52.41’N, 20°31.70’E).

**Table 1:** Localities of occurrences of *Aceria fraxiniflora* galls on green ash. Geographical coordinates, dates and name of observers for the different countries

Country	Location	Coordinates	Observers	Date
Austria	Andau	47°46.38’N, 17°01.12’E	MK	09.15.2022
Croatia	Bellye	45°36.46’N, 18°45.64’E	GyCs, AH	10.01.2022
	Zagreb	45°47.34’N, 15°59.69’E	DM, BH	09.29.2022
Czechia	Zdobín	50°24.84’N, 15°41.92’E	KH	10.17.2022
Hungary	Widespread in the whole country, recorded in 17 out of 19 counties		Korda et al. 2022	
Romania	Alesd	47°02.55’N, 22°29.01’E	GyCs, AH	08.27.2022
Serbia	Beograd	44°49.82’N, 20°22.92’E	MG	10.23.2022
	Bezdan	45°51.64’N, 18°51.50’E	GyCs, AH	10.01.2022
Slovakia	Komárom	47°45.76’N, 18°05.74’E	MK, ÁCs, DW	10.07.2022
Slovenia	Lendva	46°33.38’N, 16°26.74’E	MK, DS	03.03.2023

Abbreviations: AH (Anikó Hirka), ÁCs (Ágnes Csíszár), BH (Boris Hrasovec), DS (Dávid Schmidt), DW (Dániel Winkler), GyCs (György Csóka), KH (Karol Hradil), MG (Milka Glavendekic), MK (Márton Korda)

## Discussion

Since its first European record in Hungary in 2017, *A. fraxiniflora* has shown a rapid area expansion on both forms of the green ash, *F*. *pennsylvanica* var. *austini* and *F*. *pennsylvanica* var. *subintegerrima*. As green ash was frequently monitored before 2017 in order to record other consumers, we assume that the non-native gall mite had not been present at a detectable level before 2017. Accepting this, it is just partly possible to give an explanation for this explosive spread. The gall mites are very small and therefore can passively spread for long distance with help of the wind, road and rail traffic. Its host, green ash, is widespread and abundant in most of the region and often planted in long roadside lines, which are particularly advantageous from the point of both passive and active spread. Based on the records available so far it seems that the present distribution of *A. fraxiniflora* is concentrated in Hungary. It is partly due to our intensive monitoring, but in the same period the species has only been found sporadically in the neighbouring countries in spite of the focused search (i.e., West Romania). Regardless, given the way *A. fraxiniflora* spreads its further long-term distribution can safely be predicted.

The potential rate of spread is still hard to quantify. The first European record is from 2017 and the distribution of the species is still limited to a relatively small area. With more occurrence data from larger areas, the rate of spread will be assessed more easily. Focused monitoring of its further area expansion in Europe is called for. Population genetic studies may help locating the origin of its populations within Europe in the invaded areas and reconstructing the invasion routes.

The Central European native ash trees (*F. excelsior*, *F. angustifolia*, *F. ornus*) are frequently monitored in order to assess the impact of the ash dieback (*Hymenoscyphus fraxineus*) and detect as early as possible the appearance of the emerald ash borer (*Agrilus planipennis*) already approaching Central Europe from the direction of Russia and Ukraine. The galls of the native gall mite (*Aceria fraxinivora*) galling flowers and fruits of native ashes can be distinguished easily from the galls of *A. fraxiniflora*. As no *A. fraxiniflora* galls have ever been found on native ashes, it can be concluded that this alien species is a strict specialist of the North American ashes (*F. pennsylvanica* and *F. Americana*).

The case of the green ash and *A. fraxiniflora* supports the former experiences that the abundant occurrence of an exotic host plant will likely be followed by the appearance, establishment and spread of its also exotic consumers (Liebhold 2012, Csóka et al. 2017, Mally et al. 2021). In other words, wide scale and abundant planting of a non-native tree species will likely trigger the establishment and spread of both its alien and native consumers (herbivores and pathogens as well).

*Aceria fraxiniflora* was recorded at a wide variety of habitats of its host almost everywhere where the dioecious green ash grows. No habitat preference can be seen. Galled trees are equally found in forest habitats, along roads, in parks and any urban area. It is worth mentioning that strong intraspecific (tree-to-tree) variation in infestation rate was found (note that we only observed galls on female individuals). These differences are typical in the early expansion phase of a passively spreading species. Later the smaller infested spots will grow and merge, as it has been mentioned in case of the oak lace bug (*Corythucha arcuata*), for example (Csepelényi et al. 2017).

**Fig. 3:**
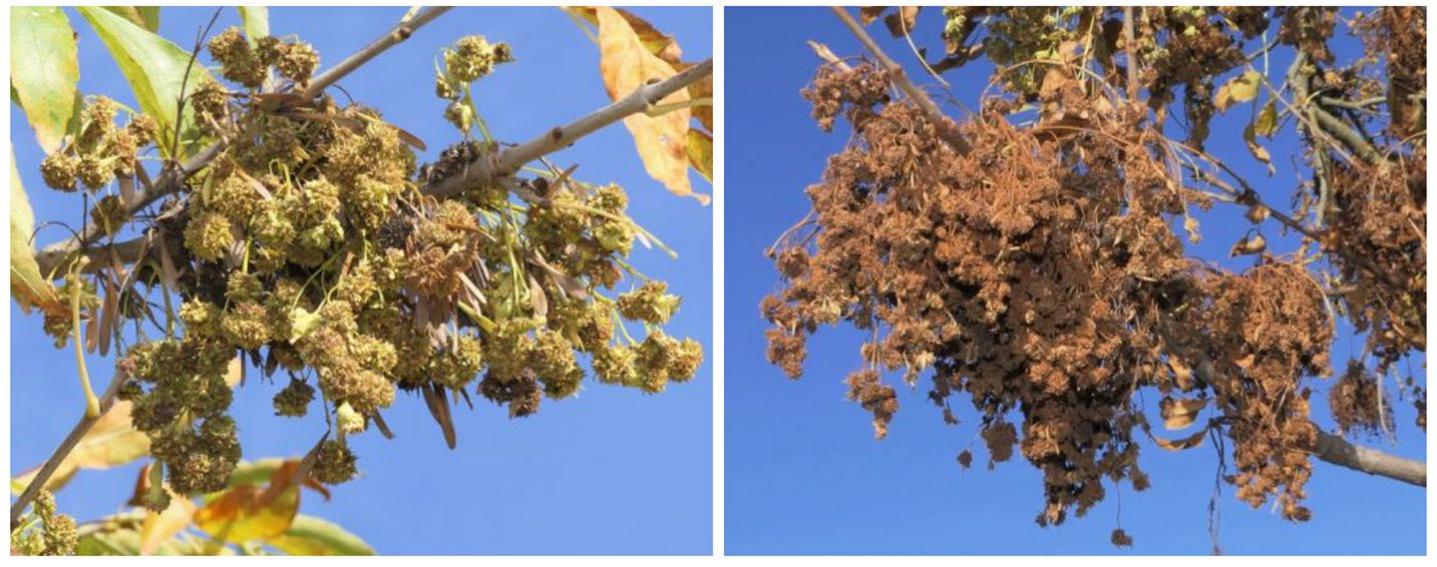
Heavy infestation on fruits of the green ash (Photos: György Csóka)

Some trees at several locations were extremely heavily infested (see Fig. 3), which raises the possibility of *A. fraxiniflora* becoming a biocontrol agent. Although the knowledge available so far is too scarce to evaluate this possibility, the impact of *A. fraxiniflora* – as specialist consumer of an invasive tree species – can be considered positive. However, its real impact on the host’s fitness should be quantified properly to evaluate its biological control potential. It should include quantifying the attack rates on inflorescences and the impact of infestation on seed germination success (lab experiments). The possibility of artificial infestation or ‘assisted spread’ of *A. fraxiniflora* (transferring inflorescences infested by mites to trees/areas still uninfested) can also be studied. These kinds of experiments may also help to reveal the real reasons of the experienced intraspecific differences in host susceptibility (random or systematic).

Several eriophyid mites are already known as biocontrol agents against weeds and semi-woody or woody plants (Smith et al. 2009). After host range testing and other preliminary studies, *Aceria genistae* was introduced to New Zealand in 2007 and to Australia between 2008 and 2010, to control *Cytisus scoparius* (broom) (Hosking et al. 2012; Paynter et al. 2012). This species appeared in the Western USA and Canada without intentional introduction and now it is considered as potential regulator of *Cytisus* (Pratt et al. 2019). Release of *Aceria angustifoliae* against *Elaeagnus angustifolia* (Russian olive) is being considered in the USA and Canada (Weyl et al. 2019; Weyl and Humair 2022). In spring 2022, the Canadian Food Inspection Agency (CFIA) approved field release of the mite in Canada.

*Aceria fraxiniflora* was most likely introduced to Europe accidentally. Since its first finding (2017, SE Hungary) it spreads rapidly and its further expansion can safely be predicted. Locally it becomes extremely abundant suggesting some kinds of potentially significant impact on its invasive host, green ash (*F. pennsylvanica*). However, there are many questions yet to be raised and answered before it can be considered a real biocontrol agent.
